# Interpretable Acoustic Features from Wakefulness Tracheal Breathing for OSA Severity Assessment

**DOI:** 10.3390/jcm15031081

**Published:** 2026-01-29

**Authors:** Ali Mohammad Alqudah, Walid Ashraf, Brian Lithgow, Zahra Moussavi

**Affiliations:** 1 Biomedical Engineering Program, University of Manitoba, Winnipeg, MB R3T 5V6, Canada; ashrafw1@myumanitoba.ca (W.A.); brian.lithgow@umanitoba.ca (B.L.); zahra.moussavi@umanitoba.ca (Z.M.); 2 Multidisciplinary Alfred Psychiatry Research Centre, Monash University, Melbourne, VIC 3004, Australia; 3 Riverview Health Center, University of Manitoba, Winnipeg, MB R3L 2P4, Canada; 4 Department of Electrical and Computer Engineering, University of Manitoba, Winnipeg, MB R3T 5V6, Canada

**Keywords:** obstructive sleep apnea, tracheal breathing sounds, machine learning, feature selection, signal processing, ensemble models, anthropometric data, explainable AI

## Abstract

**Background**: Obstructive Sleep Apnea (OSA) is one of the most prevalent sleep disorders associated with cardiovascular complications, cognitive impairments, and reduced quality of life. Early and accurate diagnosis is essential. The present gold standard, polysomnography, is expensive and resource-intensive. This work develops a non-invasive machine-learning-based framework to classify four OSA severity groups (non, mild, moderate, and severe) using tracheal breathing sounds (TBSs) and anthropometric variables. **Methods**: A total of 199 participants were recruited, and TBS were recorded whilst awake (wakefulness) using a suprasternal microphone. The workflow included the following steps: signal preprocessing (segmentation, filtering, and normalization), multi-domain feature extraction representing spectral, temporal, nonlinear, and morphological features, adaptive feature normalization, and a three-stage feature selection that combined univariate filtering, Shapley Additive Explanations (SHAP)-based ranking, and recursive feature elimination (RFE). The classification included training ensemble learning models via bootstrap aggregation and validating them using stratified k-fold cross-validation (CV), while preserving the OSA severity and anthropometric distributions. **Results:** The proposed framework performed well in discriminating among OSA severity groups. TBS features, combined with anthropometric ones, increased classification performance and reliability across all severity classes, providing proof for the efficacy of non-invasive audio biomarkers for OSA screening. **Conclusions**: TBS-based model’s features, coupled with anthropometric information, offer a promising alternative or supplement to PSG for OSA severity detection. The approach provides scalability and accessibility to extend screening and potentially enables earlier detection of OSA, compared to cases that might remain undiagnosed without screening.

## 1. Introduction

Obstructive sleep apnea (OSA) is a common yet underdiagnosed sleep-related breathing disorder affecting nearly 20% of adults in North America and linked to cardiovascular disease, hypertension, diabetes, and increased perioperative risk [1,2]. Despite its prevalence, up to 80% of cases remain undiagnosed [3], creating primary healthcare and economic burdens. OSA arises from recurrent upper airway obstruction during sleep, and its severity is classified by the apnea-hypopnea index (AHI) [4]. While polysomnography (PSG) remains the diagnostic gold standard [5], it is costly, time-intensive, and often inaccessible. Screening tools such as STOP-Bang and Berlin questionnaires provide high sensitivity but low specificity, leading to frequent misclassification of OSA status (i.e., false positives) [6,7].

Recent advances in biomedical signal analysis offer promising alternatives. For instance, tracheal breathing sounds (TBSs) recorded during wakefulness have been shown to contain distinctive acoustic markers related to upper airway physiology [8,9,10,11,12,13,14,15,16]. Studies using power spectral, bispectral, and fractal analyses, and more recently, machine learning (ML) models, have demonstrated strong potential for OSA detection [8,9,10,11,12,13,14,15,16]. However, a significant gap remains between the extraction of acoustic features and their clinical interpretation. While many studies report statistically significant differences in signal characteristics, the physiological meaning of these features and their relationships with airway mechanics, airflow resistance, and neuromuscular control remain poorly understood [17,18].

Bridging this gap is essential to translate signal-based metrics into clinically interpretable and actionable tools. This study focuses on interpreting acoustic features extracted from wakefulness TBS across different OSA severity groups. By analyzing spectral power, bispectral coupling, and fractal dimensions, we explore how acoustic signatures reflect physiological mechanisms underlying airway obstruction. This approach aims to link quantitative signal analysis with clinical interpretation, supporting the development of objective, accessible, and scalable OSA screening.

## 2. Materials and Methods

**Figure 1:**
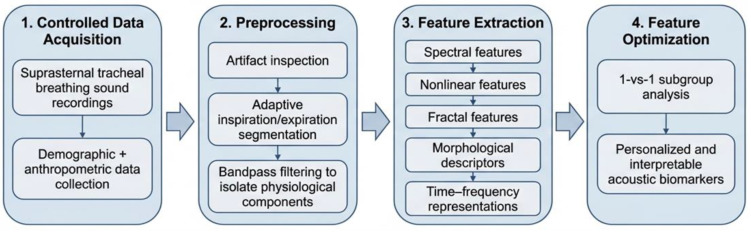
Overview of the machine learning pipeline for wakefulness-based tracheal breathing sound analysis and OSA severity classification.

In this study, we applied our previously validated workflow [8] for acquiring, preprocessing, and analyzing wakefulness tracheal breathing sounds (TBSs). Participants were recruited from individuals referred for overnight PSG, representing a clinically enriched cohort with elevated pre-test probability of OSA. Suprasternal TBS recordings were collected under controlled conditions: subjects were positioned supine and instructed to perform five full deep breaths through the nose with the mouth closed, followed by five deep breaths through the mouth while wearing a nose clip using a Sony ECM-77B, Tokyo, Japan omnidirectional condenser microphone (sensitivity: −52 dB ± 3.5 dB, frequency response: 40 Hz–20 kHz). Snoring history was not collected via subject self-report as part of the anthropometric questionnaire, and no snoring events were present in the wakeful breathing recordings analyzed in this study. Table 1 presents the distribution of subjects in the dataset by anthropometric features. Preprocessing followed our established procedures, including artifact inspection, adaptive segmentation of inspiration and expiration, and bandpass filtering to isolate physiological components. We then extracted a comprehensive set of spectral, nonlinear, fractal, morphological, and time-frequency features using the same methods detailed in [8]. These features were optimized for 1-vs-1 subgroup analyses to improve the interpretability and personalization of acoustic biomarkers. The complete workflow is summarized in Figure 1.

Preprocessing included careful inspection for background noise and vocal artifacts to be excluded, segmenting breathing sound signals into inspiratory/expiratory phases using adaptive thresholding of the log-variance envelope and Signal-to-Noise Ratio (SNR) computation, and bandpass filtering (75–3000 Hz, 4th-order Butterworth) to discard extraneous physiological and ambient signals [8,11,12]. Filtered signals were subsequently normalized using automated methods (mean-range scaling, z-score, min-max, and robust scaling) with mutual information to maximize feature-label dependency [8]. Then, a feature extraction method was applied to each processed mid-flow signal; the methodology spans multiple analytical domains, including spectral, temporal, and nonlinear analyses, as well as cross-domain analyses, ensuring a holistic, multidimensional representation of linear and nonlinear signal dynamics. The extracted features are grouped and explicitly optimized for 1-vs-1 labels [8]. This group-specific feature selection process enables the creation of personalized feature sets that enhance model robustness and improve interpretation for diagnostic and predictive applications [8]. The following features have been extracted:

- Spectral features: Power spectrum density via Welch’s method, spectral centroid, entropy, kurtosis, bandwidth, flux, and crest metrics [19].
- Bispectral features: Bootstrap-based confidence interval detection of nonstationary gaps and coupling metrics [20].
- Fractal and nonlinear features: Hurst exponent, Lyapunov exponent, Recurrence Quantification Analysis (RQA), Katz and Higuchi fractal dimensions [21,22,23].
- Wavelet and time-frequency features: Wavelet coefficients, Mel-Frequency Cepstral Coefficients (MFCCs), Constant-Q Transform statistics [24,25,26].
- Morphological features: Image-based representation of spectrogram and bispectrum (bounding box area, holes, connected components, Euler number, contrast/homogeneity/correlation/energy descriptors) [20,27,28,29].
- Time-domain metrics: Zero-crossing rate, root mean square, shimmer, jitter, and noise-to-harmonics ratio [30,31,32].

To identify stable and physiologically meaningful predictors of OSA severity, we applied the same three-stage feature selection framework described in [8], consisting of univariate statistical filtering, SHAP-based feature ranking, and RFE. Within this framework, the final feature subset was defined as the minimum number of features that preserved consistent performance across cross-validation folds while maintaining feature stability and physiological interpretability, as established in [8]. A further reduction in the feature set beyond this subset was previously shown to increase performance variability and reduce robustness; therefore, no additional feature pruning was applied in the present study. Model evaluation was conducted using a custom stratified k-fold CV scheme specifically designed to preserve the joint distribution of OSA severity labels and key anthropometric risk factors, including age, body mass index (BMI), neck circumference, sex, and Mallampati score [12]. Appendix A presents a summary of the top 35 selected features for each model, based on the extracted features.

**Figure 2:**
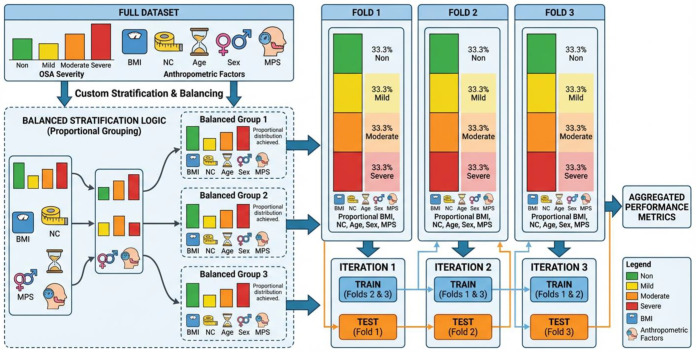
Schematic illustration of the proposed stratified *k*-fold cross-validation strategy. The full dataset is partitioned into *k* folds while preserving the proportional distribution of OSA severity classes (Non, Mild, Moderate, Severe) and key anthropometric risk factors (age, BMI, neck circumference, sex, and Mallampati score). Each fold maintains comparable joint distributions, ensuring balanced subgroup representation during training and validation and minimizing bias due to population heterogeneity.

As illustrated in Figure 2, the whole dataset is first partitioned into k folds, ensuring each fold has approximately equal representation of OSA severity classes (Non, Mild, Moderate, and Severe) and comparable distributions of the selected anthropometric variables. Rather than stratifying solely by OSA severity, the proposed strategy employs multi-criteria stratification to ensure that clinically relevant subgroups are consistently represented in both training and validation sets. This approach reduces sampling bias caused by population heterogeneity and yields a more reliable estimate of generalization performance across physiologically diverse subjects. Also, severity stratification followed standard AHI-based clinical definitions to preserve physiological granularity and enable interpretation of progressive airway dysfunction beyond binary disease detection. Table 2 shows the distribution of subjects’ anthropometric data of the k-fold splits.

**Figure 3:**
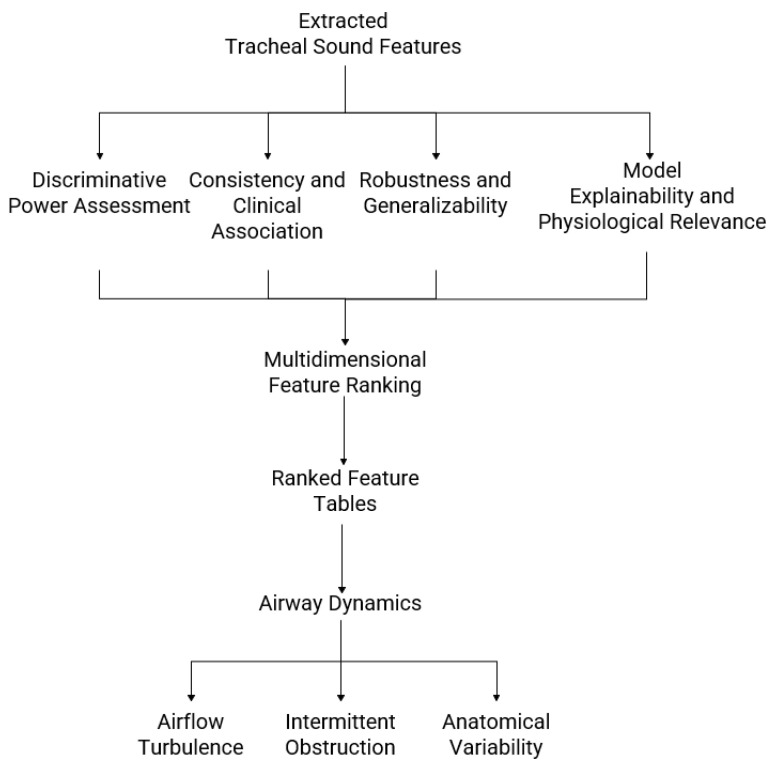
Framework for evaluating and interpreting tracheal breathing sound features in obstructive sleep apnea (OSA). Extracted acoustic and morphological features are analyzed for discriminative power, consistency with clinical variables, robustness across cross-validation folds, and model explainability. The results are integrated into a multidimensional feature ranking, producing tables, plots, and visual maps that link sound descriptors to airway dynamics, airflow turbulence, and anatomical variability across OSA severity levels.

To evaluate the discriminative power and physiological relevance of tracheal sound features, several complementary metrics were used. Area Under the Receiver Operating Characteristic (ROC) Curve (AUC) quantified each feature’s ability to distinguish between OSA severity groups, with higher values indicating stronger discrimination [33]. Pearson correlation coefficients measured the linear association between feature values and true labels, providing insight into how consistently a feature reflects the clinical outcome across cross-validation folds [34]. To assess robustness and generalizability, Absolute Delta AUC (AbsDeltaAUC) was calculated as the absolute difference between the training and test AUCs, with smaller values indicating more stable features that are less sensitive to data variability [35]. Finally, SHAP quantified each feature’s contribution to model predictions, while accounting for interactions with other features, thereby enhancing interpretability by highlighting physiologically meaningful patterns [36]. Together, these metrics enable ranking of features by both their discriminative ability and stability, supporting the identification of robust biomarkers that link acoustic and morphological descriptors to airway dynamics, airflow turbulence, and anatomical variations associated with OSA. As shown in Figure 3, the proposed feature evaluation framework integrates analyses of discriminative, correlational, stability, and explainability to identify physiologically relevant tracheal-sound features in OSA.

The final analytic framework integrates feature-level metrics, stability measures, and explainability-driven insights to produce tables and visualizations that highlight the most physiologically relevant acoustic and morphological features across OSA severity groups. Rather than focusing solely on classification performance, the analysis emphasizes how each feature contributes to model predictions and relates to underlying airway physiology. Ranked lists based on AUC [33], fold-wise stability (AbsDeltaAUC) [35], and SHAP values [36] provide a multidimensional perspective on feature importance, while correlation analyses link these features to clinical and anthropometric variables, including AHI, neck circumference (NC), and Mallampati Score (MPS) [34]. Image-based and spectro-temporal feature maps illustrate changes in sound texture, frequency patterns, and event shapes, revealing airflow turbulence, intermittent obstruction, and variations in airway mechanics. By combining quantitative metrics with visual interpretations, this framework transforms raw signal descriptors into clinically meaningful biomarkers, enhancing understanding of upper-airway dynamics, airflow irregularities, and anatomical risk factors associated with OSA severity [37].

## 3. Results

This section presents the key findings from the feature extraction and selection pipeline, highlighting the most discriminative tracheal-breathing-sound features for OSA severity classification. Analyses were conducted across six 1-vs-1 base models (Non-OSA vs. Mild, Non-OSA vs. Moderate, Non-OSA vs. Severe, Mild vs. Moderate, Mild vs. Severe, Moderate vs. Severe) and three folds of a custom stratified cross-validation, designed to preserve the joint distribution of severity groups and key anthropometric factors. Feature importance was assessed using both correlation-based ranking and SHAP values to identify consistently essential features. The top selected features for each model are detailed in Appendix A.

**Figure 4:**
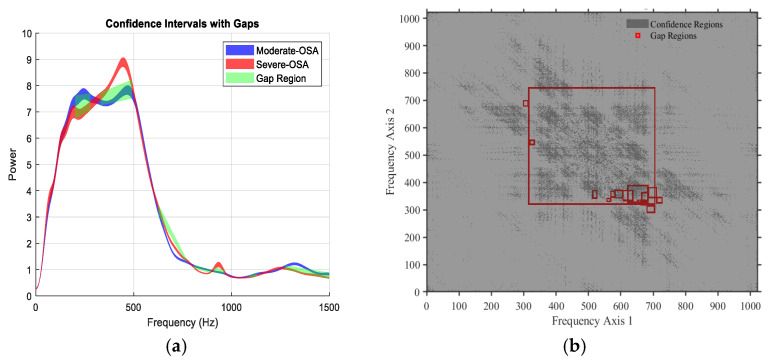
Representative examples of gap regions identified between two classes based on non-overlapping 95% confidence intervals. (**a**) Power spectral density (PSD): the green shaded areas indicate frequency bands where the 95% confidence intervals of the average PSD for Moderate-OSA and Severe-OSA do not overlap, defining statistically significant spectral gaps between the two classes. (**b**) Bispectrum: red boxes indicate time–frequency regions in the bispectral domain where the 95% confidence intervals of the averaged bispectrum for Moderate-OSA and Severe-OSA do not overlap. The bispectrum, a third-order spectral measure, captures quadratic phase coupling and nonlinear interactions between frequency components; gap regions therefore indicate class-specific differences in nonlinear spectral coupling.

The models and selected features are empirical; their exact frequency bands or characteristics may differ for other datasets depending on the sensor used (e.g., different microphones). To keep feature names readable, we have categorized them by main characteristics, such as spectral or bispectral features, breathing type (mouth or nose), and phase (inspiration or expiration). The frequency regions from which the features were extracted are based on the 95% confidence interval of the training set, as proposed in our previous work [8]. For example, Bispectral_Centroid_Mean represents the mean bispectral energy centroid across all breathing conditions. Similarly, Spectral_Skewness_Mouth_Inspiration captures the skewness of the spectral distribution during mouth inspiration, and Spectral_FrequencyRatio_Mouth_Expiration represents the frequency ratio feature during mouth expiration. The detailed definitions of these features, including what a bounding box (BBox) is, the specific coordinates or frequency/time ranges, and the corresponding breathing conditions, are provided elsewhere (e.g., in a footnote, appendix, or table legend). This approach ensures that the main text remains readable while maintaining reproducibility and technical clarity. This general naming approach avoids dataset-specific details, makes the features more interpretable for readers outside the team, and preserves the essential information on how each feature was derived (Figure 4).

For detailed clinical interpretation of top features, a specific appendix (Appendix B) is dedicated to these details, while for detailed comparison-specific results, all supporting tables and figures are provided in the appendices: Appendix C (Non-OSA vs. Mild OSA), Appendix D (Non-OSA vs. Moderate OSA), Appendix E (Non-OSA vs. Severe OSA), Appendix F (Mild vs. Moderate OSA), Appendix G (Mild vs. Severe OSA), and Appendix H (Moderate vs. Severe OSA). Each appendix includes the top-ranked features by test AUC, the most stable features by AbsDeltaAUC, and the strongest anthropometric- and AHI-associated features for the corresponding comparison. Higher AUC values indicate greater discrimination between the two severity groups. In contrast, smaller AbsDeltaAUC values suggest more consistent feature performance across cross-validation folds, reflecting reduced variability in AUC estimates across data partitions. Correlation analyses with anthropometric variables and AHI provide additional insight into potential physiological relevance and relationships with clinical severity. The following subsections focus on the clinical interpretation of the most consistently supported features across these comparisons.

### 3.1. Clinical Interpretation of Top Features

To provide a physiologically grounded interpretation of the observed acoustic differences, we adopt a Structure–Function–Symptom framework. In this narrative, anatomical and structural characteristics of the upper airway (Structure), such as tissue compliance, airway narrowing, and fat deposition, influence airflow behavior during breathing (Function), including turbulence, nonlinear coupling, and ventilatory instability. These functional alterations manifest clinically as differences in apnea–hypopnea burden and disease severity (Symptom), quantified by the AHI. The following interpretations therefore explain how each significant acoustic feature reflects a structural-functional pathway underlying OSA progression.

This subsection provides a clinical interpretation of the most discriminative features, prioritized based on agreement across AUC ranking, AbsDeltaAUC, and SHAP importance. The goal is to link key acoustic and spectro-temporal descriptors to potential physiological and airflow changes associated with early manifestations of sleep-disordered breathing. Table 3 shows an overview of the clinical interpretation of features across different models. In contrast, more detailed feature-by-feature interpretations are provided in Appendix B. Collectively, this analysis facilitates a clearer understanding of how specific acoustic patterns may reflect underlying upper airway dynamics and disease progression. Furthermore, aligning model-derived features with known clinical mechanisms enhances the interpretability and translational relevance of the proposed framework.

### 3.2. Top-Ranked Features

The top 10 features, identified by their overall average rank across both correlation- and SHAP-based ranking methods, are presented in Table 4. These features consistently demonstrated high importance in distinguishing between different OSA severity groups. The top features include a mix of spectral, temporal, and morphological characteristics of the tracheal breathing sounds, as shown in Table 4. Notably, features related to spectral bandwidth (range of frequencies contributing to the signal), texture energy (quantifies uniformity and repetitiveness of bispectral patterns), spectral flux (measures frame-to-frame changes in the power spectrum), and statistical moments (mean, standard deviation, kurtosis, skewness) consistently appear among the most essential features. These features are robust across cross-validation folds and severity comparisons, indicating that they reliably capture physiologically relevant changes in airflow dynamics and turbulence. These features capture various aspects of the sound signal, including its frequency distribution, temporal dynamics, and overall intensity and complexity.

### 3.3. Feature Stability Across Folds and Models

The top 10 most stable tracheal breathing sound features were identified based on the lowest absolute differences between training and testing Area Under the Curve (AUC) values, as shown in Table 5. These features demonstrate minimal variability across different data splits, highlighting their robustness and consistency in discriminating OSA severity. Lower absolute Delta AUC values indicate that the predictive power of these features is reliably maintained across training and test datasets. In contrast, lower absolute Delta Corr values reflect more consistent correlations between the features and clinical measurements, indicating stable physiological relevance across folds. Collectively, these metrics suggest that the selected features are both robust and physiologically meaningful, making them strong candidates for inclusion in predictive models and clinical decision-support systems.

### 3.4. Correlation with Anthropometric Data

Strong associations were observed between several extracted tracheal-sound features and key clinical anthropometric measurements relevant to OSA, including BMI, NC, Sex, and MPS. These relationships, summarized in Table 6, highlight the clinical relevance of the acoustic and spectro-temporal descriptors identified in this study. The table emphasizes the features most strongly correlated with anthropometric parameters, providing insight into the physiological underpinnings of OSA severity and supporting their potential utility in predictive modeling.

Pearson correlation coefficients between acoustic features and anthropometric variables were computed independently within each cross-validation fold, rather than on pooled data. For each severity comparison, correlations were calculated using the fold-specific data subset, and the corresponding fold index is explicitly reported in Table 6.

These correlations were used exclusively for interpretability and physiological analysis and did not influence model training, feature selection, or classifier optimization. As such, they should not be interpreted as estimates of generalization performance, but rather as indicators of strong fold-specific associations between acoustic characteristics and anthropometric measures.

## 4. Discussion

This study aimed to identify interpretable features from wakefulness tracheal breathing sounds that are clinically relevant for assessing OSA severity. The consistent emergence of specific features across different models and folds, coupled with their stability and correlation with anthropometric data, underscores their potential as robust biomarkers for OSA [8,9,10,11,12,13,17,18,38].

### 4.1. Clinical Relevance of Key Features

Interpreting acoustic biomarkers through a structure–function–symptom lens enables a mechanistic understanding of how anatomical vulnerability of the upper airway translates into altered airflow dynamics and ultimately manifests as increasing OSA severity. OSA manifests through complex interactions between the upper airway anatomy, airflow turbulence, and respiratory control. Identifying features from tracheal breathing sounds recorded whilst awake that reliably reflect these physiological processes is crucial for non-invasive assessment. This section focuses on clinically meaningful indicators of OSA severity [39,40,41].

#### 4.1.1. Non-OSA vs. Mild-OSA

From a structure–function–symptom perspective, early anatomical vulnerability of the upper airway (Structure), including mild tissue compliance and partial narrowing, leads to subtle functional airflow disturbances (Function), characterized by intermittent turbulence and disrupted nonlinear airflow–tissue coupling during inspiration. These functional alterations manifest clinically (Symptom) as mild elevations in AHI without sustained airway obstruction. The distinction between Non-OSA and Mild-OSA is characterized by the emergence of subtle yet consistent early signs of the upper airway instability during breathing whilst awake. These changes are captured by features such as MouthInspiration_Range_FreqSkewness, Average_BBox_TextureEnergy, and Average_BBox_FrequencyCentroidX, which quantify shifts in spectral energy, disruption of structured bispectral coupling, and changes in dominant frequency interactions, respectively (see Appendix B.1 for full feature definitions and physiological interpretation). The observed patterns indicate a transition from predominantly laminar airflow toward intermittently turbulent inspiratory flow, consistent with early upper-airway collapsibility and soft-tissue vibration [38,40,42]. These changes suggest the onset of periodic flow limitation without sustained obstruction, aligning with early physiological manifestations of mild OSA described in prior studies [16,41].

Overall, mild OSA is marked not by significant increases in breathing sound intensity but by early disruption of airflow regularity and spectro-temporal organization, as captured by these spectral and bispectral features. This finding supports the concept that *the earliest stage of OSA manifests primarily as micro-instability and intermittent turbulence rather than overt obstruction, reinforcing the value of wakeful acoustic markers for early detection* [16,43,44].

#### 4.1.2. Non-OSA vs. Moderate-OSA

Within the structure–function–symptom framework, progressive anatomical narrowing and reduced airway stiffness (Structure) produce sustained airflow limitation and elevated inspiratory effort (Function), resulting in prolonged turbulent breathing events. Clinically (Symptom), these changes correspond to a clear increase in AHI and more frequent obstructive events, consistent with moderate OSA. In contrast to mild OSA, the transition from Non-OSA to Moderate-OSA reveals a clear escalation in airflow disturbance and respiratory effort. These changes are captured by features such as Average_Range_Maximum, Average_BBox_BoundingBoxDiagonal, Average_Range_MeanPower, and Average_BBox_ConnectedComponents, which quantify peak breathing sound energy, expansion of nonlinear bispectral interactions, overall sound intensity, and fragmentation of coupling patterns, respectively (see Appendix B.2 for full feature definitions and physiological interpretation). The results demonstrate stronger, more sustained turbulent breathing events, reflecting prolonged partial airway collapse and increased inspiratory drive [13,39,45]. Breathing sounds become more energetic and fragmented, consistent with repetitive cycles of obstruction and compensatory recovery.

These acoustic characteristics indicate that *moderate OSA is physiologically defined by persistent airflow instability rather than isolated abnormalities.* The increased duration, intensity, and fragmentation of breathing events align with established descriptions of heightened airway collapsibility and more frequent arousal-related breathing responses in moderate disease [46,47,48].

#### 4.1.3. Non-OSA vs. Severe-OSA

Structurally, severe OSA is characterized by pronounced upper-airway collapsibility and reduced neuromuscular compensation. Functionally, this leads to chaotic airflow, repeated collapse–reopening cycles, and highly nonlinear breathing dynamics. These functional disturbances manifest clinically as high AHI values (Symptom), reflecting frequent apneic and hypopneic events. *Severe OSA exhibits a markedly distinct acoustic phenotype, dominated by chaotic, high-energy, and highly irregular breathing patterns.* These changes are captured by features such as Average_BBox_MeanValue, Average_BBox_TextureEnergy, Average_BBox_FractalDimension, Average_BBox_EnergyValue, and Average_BBox_KurtosisValue, which quantify average sound intensity, heterogeneity of bispectral coupling, complexity of local patterns, total sound energy, and prevalence of abrupt or impulsive events, respectively (see Appendix B.3 for complete feature definitions and physiological interpretation). The results indicate frequent and intense airflow collapse followed by forceful recovery breaths, producing complex and impulsive acoustic events across a broad frequency range [12,38,40]. The pronounced variability and structural disruption observed are consistent with unstable ventilatory control and recurrent airway obstruction.

Physiologically, these findings reflect deep upper-airway collapsibility, exaggerated negative pressure swings, and repeated collapse–reopening cycles characteristic of advanced OSA [41,46,49]. The elevated complexity and unpredictability of the acoustic patterns are in line with prior reports linking severe disease to chaotic airflow and disordered breathing mechanics [50,51,52].

#### 4.1.4. Mild-OSA vs. Moderate-OSA

In structure–function–symptom terms, the transition from mild to moderate OSA reflects worsening anatomical compromise of the airway (Structure), which shifts airflow behavior from intermittent to persistent instability (Function). This progression manifests clinically (Symptom) as a sustained increase in AHI and reduced effectiveness of compensatory airway control. The progression from mild to moderate OSA represents a shift from intermittent airflow disturbance to more persistent and structurally disruptive obstruction. These changes are captured by features such as Average_BBox_MeanValue, Average_BBox_TextureEnergy, Average_BBox_FractalDimension, Average_BBox_EnergyValue, and Average_BBox_KurtosisValue, which quantify average sound intensity, heterogeneity of bispectral coupling, complexity of local patterns, total sound energy, and the prevalence of sharp or impulsive events, respectively (see Appendix B.4 for complete feature definitions and physiological interpretation). The results indicate increasing turbulence during both inspiration and expiration, accompanied by broader spectral involvement and greater fragmentation of breathing sounds [12,38,44,53]. This suggests that airflow irregularities are no longer isolated but sustained throughout the respiratory cycle.

Clinically, this transition reflects worsening airway collapsibility and reduced effectiveness of neuromuscular compensation during wakefulness. Moderate OSA therefore emerges as a state in which airflow instability becomes chronic rather than episodic, consistent with physiological models of disease progression [9,17,40,50].

#### 4.1.5. Mild-OSA vs. Severe-OSA

Here, structural airway vulnerability becomes dominant (Structure), overwhelming compensatory mechanisms. Functionally, this produces highly variable, noisy, and energetically intense airflow patterns. Clinically (Symptom), these effects correspond to severe OSA, marked by large AHI values and pronounced breathing instability. Comparisons between mild and severe OSA highlight a pronounced escalation in airflow irregularity, respiratory effort, and acoustic unpredictability. These changes are captured by features such as MouthInspiration_BBox_FrequencyCentroidX, Average_Average_BBoxes_Entropy, Average_Range_RMS, Average_BBox_EnergyValue, and MouthExpiration_Range_SpectralEnergy, which quantify shifts in dominant frequencies, overall entropy of bispectral patterns, amplitude variability, total sound energy, and broadband spectral energy, respectively (see Appendix B.5 for complete feature definitions and physiological interpretation). The results reveal prolonged and noisy inspiratory phases, increased breath-to-breath variability, and intense turbulent bursts extending into expiration [38,44,45]. These patterns indicate a breakdown of compensatory airway control mechanisms that remain partially effective in mild disease.

*From a physiological standpoint, severe OSA is characterized by loss of airflow stability, where collapsibility dominates over neuromuscular control.* The marked increases in variability and turbulence observed align with descriptions of unstable ventilatory control and repeated collapse–recovery dynamics in severe disease [18,38,44,48].

#### 4.1.6. Moderate-OSA vs. Severe-OSA

From a structure–function–symptom standpoint, severe OSA represents a qualitative shift rather than a linear extension of moderate disease: deeper structural collapse and airway instability (Structure) lead to near-chaotic airflow dynamics (Function), which clinically manifest (Symptom) as extreme AHI values and frequent obstructive episodes. The transition from moderate to severe OSA is marked by a qualitative shift from structured instability to near-chaotic airflow dynamics. These changes are captured by features such as Average_BBox_MedianValue, Average_BBox_IQRValue, Average_BBox_EnergyValue, Average_BBox_KurtosisValue, Average_BBox_Compactness, Average_BBox_StdValue, and Average_BBox_EntropyValue, which quantify overall breathing sound intensity, central and total variability, abrupt peaks, diffusion of high-intensity regions, dispersion, and randomness of airflow-related acoustic patterns, respectively (see Appendix B.6 for full feature definitions and physiological interpretation). The results indicate greater breath-to-breath variability, stronger and more erratic respiratory effort, and increasingly diffuse turbulent sound patterns [39,41,48]. Acoustic events become less compact and more topologically complex, reflecting deeper and more frequent airway collapse.

These findings suggest that *severe OSA represents not merely an amplification of moderate disease but a distinct physiological regime characterized by unpredictable airflow, unstable arousal responses, and diminished airway resilience* [18,38,44,50,54]. This distinction supports the clinical importance of separating moderate and severe OSA in severity stratification and management.

#### 4.1.7. Physiological Themes Across Models

Across severity comparisons, certain recurring acoustic patterns reflect underlying physiological mechanisms of OSA. By examining features related to turbulence, airflow complexity, variability, and energy, we can identify consistent markers of airway instability, vibration, and compensatory respiratory effort. The following themes summarize how these features may collectively capture the progression of OSA.

**Escalating turbulence, bandwidth, and centroid shifts** correspond to rising Reynolds number and more pronounced vibration/snoring as the airway narrows [44].
**Event complexity (diagonals, perimeters, shape metrics):** track segmented, irregular airflow fragments as OSA severity increases [43].
**Variability (Interquartile range (IQR), Standard Deviation (SD), entropy)** reveals unstable ventilatory control, frequent arousals, and abrupt collapse–recovery dynamics [43].
**Amplitude/energy (mean, RMS, total)** reflect increasing respiratory effort, loud post-obstructive inspiration, and compensatory surges in disease progression.

Each feature thus provides a physiologic aspect into how OSA disrupts the upper airway patency, generates turbulence and vibration, and drives instability and variability across both models [43,44].

### 4.2. Rationale for Multi-Class OSA Severity Stratification

While binary OSA classification (OSA vs. non-OSA) is common in screening-oriented studies, it does not capture the progressive and heterogeneous nature of the upper-airway dysfunction. Clinically defined AHI severity categories (mild, moderate, and severe) reflect distinct physiological states, including differences in airway collapsibility, airflow turbulence, ventilatory compensation, and symptom burden.

In this study, several acoustic features exhibited nonlinear or stage-specific behavior across severity levels, particularly in comparisons involving mild-to-moderate and moderate-to-severe transitions. Collapsing these groups into a binary framework would obscure intermediate phenotypes and reduce sensitivity to early or transitional disease mechanisms. By adopting a four-class framework, the proposed model preserves physiologically meaningful distinctions and enables severity-aware interpretation aligned with clinical risk stratification, perioperative assessment, and treatment decision-making.

### 4.3. Physiological and Clinical Interpretation of Feature Linkage to Severity

The high-ranking and stable features are not merely statistical constructs; they are direct acoustic manifestations of the anatomical and functional changes associated with progressive OSA severity. Tracheal sounds are generated by turbulent airflow, and their characteristics are susceptible to subtle changes in airway geometry, collapsibility, and compensatory respiratory effort, even during wakefulness [8,11].

Although all acoustic recordings in this study were obtained whilst awake under quiet breathing conditions and therefore do not contain snoring events, snoring history remains an important symptom associated with OSA severity. Chronic snoring reflects repetitive vibration of upper-airway soft tissues during sleep, which has been hypothesized to contribute to long-term structural changes such as tissue remodeling, inflammation, or altered compliance. These chronic structural modifications may persist beyond sleep and subtly influence airflow behavior and tracheal breathing acoustics even during wakefulness. Consequently, while the extracted acoustic features do not represent snoring sounds per se, they may indirectly reflect cumulative airway alterations associated with both OSA severity and a history of habitual snoring. Future studies incorporating quantitative snoring indices alongside wakeful and sleep-state recordings may further clarify this relationship.

#### 4.3.1. Acoustic Signatures of Airway Chaos and Ventilatory Effort (AHI Correlation)

The correlations with the AHI provide the most direct clinical linkage. The following summarizes the correlations of features with AHI:

- Decreased Texture Energy (Acoustic Disorganization): The feature exhibits the strongest negative correlation with OSA severity. Texture Energy is a quantitative measure of the uniformity and repetitiveness of local patterns in a spectrogram, computed by summing the squared values of the co-occurrence or filtered spectrogram matrix, reflecting how consistent and regular the acoustic structure is. As severity increases, the pharyngeal airway becomes intrinsically more compliant and prone to intermittent vibration and collapse, leading to flow separation and highly random, broadband turbulence. This shift from structured, laminar-like noise to chaotic, broadband turbulence disrupts the consistency of the spectrogram, resulting in a significant decrease in texture energy. This feature, therefore, serves as a powerful acoustic marker of increasing pharyngeal instability and vulnerability [39,42].
- Increased Skewness (Compensatory Drive): Conversely, the high positive correlation (r ≈ 0.99) between spectral skewness and OSA severity indicates systematic changes in the distribution of sound amplitude. Positive skewness signifies a heavier tail toward high-amplitude values. Physiologically, this represents the subject’s increased reliance on intermittent, high-force maneuvers (such as a forceful, highly turbulent inhalation or a loud snort/gasp) to maintain adequate flow against increasing pharyngeal resistance. Clinically, this feature is an acoustic signature of heightened respiratory drive and compensatory effort, which scales directly with disease burden [41,45].

#### 4.3.2. Morphological and Spectral Markers of Flow Limitation and Airway Dynamics

The consistently top-ranked spectral and morphological features provide a detailed view of the fluid dynamics within the compromised airway.

- **Spectral Bandwidth and Flux (Venturi Effect):** These features are crucial markers of dynamic flow behavior. Airflow acceleration through a narrow, compliant pharyngeal segment (the site of flow limitation, a manifestation of the Venturi effect) generates high-velocity jets. The high spectral flux reflects the rapid, transient changes in the power spectrum as these turbulent jets form and dissipate during the breathing cycle. In contrast, increased bandwidth reflects a broader spread of acoustic energy across frequencies. Together, these changes are consistent with the presence and severity of flow-limiting segments, where the degree of narrowing modulates the strength and spectral extent of turbulent eddies [40,55].
- **Fractal Dimension and Complexity (Non-linear System Behavior):** The high-ranking fractal dimension quantifies the non-linear complexity of the signal. Increased airway resistance and turbulence are hallmarks of a system pushed toward instability. A higher fractal dimension suggests a highly complex, chaotic, and less predictable airflow pattern, aligning with established non-linear control theory, which views the respiratory system as operating close to a chaotic bifurcation point [50,51].

#### 4.3.3. Validation Through Established Anatomical Risk Factors

The extremely high correlations between specific features and anthropometric measures provide vital proof of concept: the acoustic features are not just abstract discriminators but directly encode the physical risk factors [12,56,57]. Peak Intensity is the near-perfect correlation between Neck Circumference (NC), which is physiologically profound. It is a validated proxy for fat deposition and reduced pharyngeal tissue stiffness. This deposition not only narrows the airway but also influences sound wave propagation. The high peak acoustic value in expiration is a measurable outcome of a sound wave propagating through a physically constrained, often partially occluded, and highly compressible tissue structure. At the same time, Entropy is the strong correlation between (a proxy for systemic obesity and increased soft tissue mass) and links overall body habits to the acoustic randomness and disorganization of the expired airflow pattern.

The combined evidence from correlation, feature stability, and anthropometric validation strongly supports the use of wakefulness features for the objective and clinically meaningful assessment of severity [12,56,57].

### 4.4. Correlation with Anthropometric Data

The strong correlations observed between several tracheal sound features and anthropometric data further strengthen their clinical utility [12,17]. For example, features such as Mouth Expiration_BBox_650_15_1_0_peakValue, which correlate strongly with Neck Circumference (NC), and Mouth Expiration_BBox_155_2_0_0_entropyValue, which correlate strongly with BMI, are particularly noteworthy. NC and BMI are well-established risk factors and indicators of OSA severity. The direct relationship between these anthropometric measures and specific acoustic features suggests that the structural characteristics of the upper airway, influenced by body posture, are reflected in tracheal breathing sounds [15,58]. This provides a mechanistic link between anatomical predispositions to OSA and the acoustic manifestations captured by our features. Similarly, correlations with the Mallampati Score (MPS), an indicator of the oral cavity and pharyngeal space, further support the idea that the airway’s physical configuration influences sound production during breathing [47]. Although some fold-wise correlations approached unity, these values reflect strong associations observed within specific severity contrasts and folds and do not imply model overfitting, as correlation analysis was conducted independently of the predictive learning pipeline.

These findings suggest that wakefulness tracheal breathing sounds contain rich information reflecting the physiological state of the upper airway. The ability of these features to discriminate OSA severity during wakefulness, their stability across different models and folds, and their strong correlations with established anthropometric risk factors are particularly significant. This offers a non-invasive, convenient, and potentially cost-effective method for screening and monitoring [9,44,59]. The interpretability of these features allows for a deeper understanding of the underlying mechanisms of OSA, moving beyond black-box model predictions [9,44,59]. This interpretability is crucial for clinical acceptance and for guiding future research into targeted interventions.

### 4.5. Alignment with Prior Wakefulness-Based OSA Studies

The present findings align strongly with and extend our group’s previous works [12,17]. In the 2018 study [17], the authors demonstrated that spectral, bispectral, and fractal features extracted from tracheal sounds could effectively differentiate OSA from non-OSA subjects, achieving classification accuracies of approximately 70–75% and ROC values of 0.73–0.80. They further observed that mouth-inspiratory features provided the highest discrimination power and that several acoustic descriptors were only weakly influenced by anthropometric variability, such as body mass index (BMI) or neck circumference (NC). In the other 2019 study [12], they reinforced these results using a larger dataset of 199 subjects. It confirmed that combining tracheal sounds with anthropometric features increased diagnostic performance to 81.4% accuracy (sensitivity = 82.1%, specificity = 80.9%).

Consistent with prior findings, our results achieved diagnostic performance comparable to or superior to that of previous models, yielding AUC values ranging from 0.86 to 0.97 across multiple OSA severity levels. The strongest predictive power was again observed for mouth-inspiratory and low-frequency components (150–450 Hz), which exhibited elevated spectral energy and distinct morphological patterns in moderate and severe OSA subjects. Correlation analysis in our dataset similarly showed significant relationships between acoustic features and anthropometric markers, including BMI (r = 0.52–0.75) and NC (r = 0.47–0.72), corroborating earlier physiological interpretations of airway constriction and turbulence during inspiration.

Beyond confirming the earlier results, the present study extends the prior research in three significant ways:

1. Instead of a binary OSA vs. non-OSA classification, our framework performs multi-level severity stratification (non-OSA, mild, moderate, and severe), offering finer clinical granularity.
2. We introduce novel morphological and time–frequency gap descriptors, extracted from harmonic–percussive (HP) decompositions and spectrogram bounding boxes, which capture airway-specific acoustic signatures not examined in previous work.
3. Our use of ensemble-based models with SHAP explainability provides transparent quantification of feature contributions and robustness validation (Abs ΔAUC < 0.04 across folds), establishing reproducibility across subjects and folds.

Together, these advances confirm and expand on the foundational evidence presented previously by our group [12,17], demonstrating that wakefulness-based tracheal breathing sounds, combined with simple anthropometric measures, constitute a physiologically meaningful, non-invasive, and reproducible tool for OSA detection and severity classification.

### 4.6. Comparison with Other Awake Screening Modalities

To contextualize the proposed methodology, it is necessary to benchmark it against the full spectrum of screening modalities whilst awake. While questionnaires (e.g., STOP-Bang) are ubiquitous due to their zero-cost administration [60], they are hindered by low specificity (often <40%), leading to high false-positive rates [60]. Facial image analysis offers a non-contact alternative, but current methods often plateau at approximately 70% accuracy or require large, balanced datasets to avoid demographic bias [61]. Functional methods such as Negative Expiratory Pressure (NEP) [62] and Acoustic Pharyngometry [63] offer high accuracy by directly measuring airway collapsibility and geometry. However, these techniques often require specialized equipment and strictly controlled protocols, reducing their utility for rapid, large-scale screening compared to microphone-based approaches [12]. Speech analysis, while similar in modality to tracheal breathing sounds analysis [64], often relies on complex phonetic tasks and has shown lower accuracy and variable specificity depending on the features used.

Table 7 presents a comprehensive quantitative comparison between the proposed framework and representative studies across five distinct wakefulness-based modalities. To ensure a fair comparison, we focus on dataset size, task formulation, and performance metrics (Sensitivity/Specificity). This comparison highlights that while functional tests (NEP/Pharyngometry) offer high precision, they lack the portability of acoustic methods. Conversely, while questionnaires are portable, they lack the diagnostic accuracy of the proposed method.

### 4.7. Limitations and Future Work

Although the proposed framework achieved high interpretability, several technical limitations merit consideration. The analysis was performed on data collected from a single clinical site [8,11,12], which may limit the generalizability of the results to broader populations with differing acoustic environments, recording hardware, and demographic characteristics. Secondly, the sound recordings were conducted by a high-end Sony microphone. The selected features may change (although not significantly) depending on the sensor used. Future studies should incorporate multi-center datasets and cross-device validation to ensure robustness under real-world variability. Additionally, while the stratified k-fold design effectively balanced anthropometric covariates, the current results were based on a finite number of wakefulness recordings per subject, restricting the temporal representation of respiratory dynamics. Expanding the framework to include multi-cycle, sleep-stage-specific, or longitudinal tracheal sound data would enable modeling of disease progression and treatment response. Despite these constraints, the consistent feature stability and explainable ensemble structure provide a strong foundation for advancing automated, non-invasive OSA assessment.

In addition to these technical considerations, the study cohort consisted of individuals referred to overnight PSG and therefore represents a clinically enriched population with a higher pre-test probability of OSA than general or primary-care populations. This enrichment may lead to optimistic estimates of discrimination metrics, such as AUC, compared with deployment in lower-risk settings, and may particularly affect positive predictive value when disease prevalence is lower. However, the primary objective of this work was not to estimate population-level screening accuracy but to identify robust, physiologically interpretable acoustic biomarkers of OSA severity under controlled clinical conditions. These biomarkers reflect underlying airway dynamics and anatomical vulnerability, which are expected to generalize beyond referral-based cohorts.

In practical deployment, the proposed framework is best positioned as a first-line risk stratification or prioritization tool rather than a standalone diagnostic test. In primary-care or community settings, it could be used to identify individuals who would benefit most from expedited PSG, thereby improving resource allocation and reducing diagnostic delays. Future studies will focus on validating the framework in lower-prevalence populations, including primary-care and community-based cohorts, and on recalibrating decision thresholds to account for differences in disease prevalence and pre-test probability. Despite these constraints, the consistent feature stability and explainable ensemble structure provide a strong foundation for advancing automated, non-invasive OSA assessment.

## 5. Conclusions

In conclusion, this research successfully established an interpretable, machine-learning-driven framework that uses wakefulness tracheal breathing sounds as objective, severity-stratifying biomarkers for Obstructive Sleep Apnea. By identifying and validating stable acoustic features, texture energy, spectral bandwidth, and fractal dimension that exhibit strong mechanistic correlations with established anatomical risk factors, we have acoustically encoded the underlying physiological vulnerability of the upper airway. This interpretability moves beyond black-box diagnostics, offering clinicians clear, physiological correlates for disease progression. These findings represent a decisive step toward developing a non-invasive, cost-effective, and highly accessible screening tool, which is critically needed for timely diagnosis, perioperative risk stratification, and scalable long-term management in clinical settings globally.

## Figures and Tables

**Table 1:** Participants’ Severity Groups and Anthropometric Information. AHI: apnea–hypopnea index, BMI: body mass index, NC: neck circumference, MPS: Mallampati score, M/F: male/female.

Severity Group	Number of Subjects	AHI	Sex	Age	NC	BMI	MPS
Non-OSA	74	1.2 ± 1.3	29 M, 45 F	46.8 ± 12.9	38.8 ± 4.0	30.6 ± 6.2	41 (1), 19 (2), 6 (3), 8 (4)
Mild	35	8.7 ± 2.6	21 M, 14 F	52.3 ± 11.6	42.1 ± 6.5	34.3 ± 8.4	18 (1), 6 (2), 9 (3), 1 (4)
Moderate	50	21.5 ± 4.2	36 M, 14 F	54.7 ± 11.3	43.1 ± 3.4	33.8 ± 6.4	17 (1), 17 (2), 8 (3), 8 (4)
Severe	40	69.5 ± 33.3	30 M, 10 F	48.9 ± 11.1	45.3 ± 3.6	39.7 ± 8.7	5 (1), 13 (2), 14 (3), 8 (4)

**Table 2:** Participants’ Severity Groups and Anthropometric Information for K-folds. AHI: apnea–hypopnea index, BMI: body mass index, NC: neck circumference, MPS: Mallampati score, M/F: male/female.

Severity Group	Fold	Number of Subjects	AHI	Sex	AGE	BMI	NC	MPS
Non-OSA	1	23	0.6 ± 0.8	10 M, 13 F	44.9 ± 12.1	29.2 ± 4.7	38.0 ± 4.7	12 (1), 7 (2), 1 (3), 3 (4)
	2	27	1.1 ± 1.3	10 M, 17 F	45.7 ± 12.1	32.3 ± 7.6	39.2 ± 4.3	12 (1), 9 (2), 4 (3), 2 (4)
	3	24	1.8 ± 1.3	9 M, 15 F	50.0 ± 14.3	30.0 ± 5.8	39.0 ± 2.9	17 (1), 3 (2), 1 (3), 3 (4)
Mild	1	16	8.7 ± 2.4	10 M, 6 F	50.9 ± 12.5	36.6 ± 9.9	43.5 ± 5.3	7 (1), 1 (2), 7 (3), 1 (4)
	2	10	8.6 ± 2.2	5 M, 5 F	51.3 ± 11.6	31.8 ± 8.4	38.5 ± 8.8	6 (1), 1 (2), 2 (3), 1 (4)
	3	9	8.8 ± 3.5	6 M, 3 F	56.0 ± 10.3	33.0 ± 4.2	43.4 ± 2.7	5 (1), 4 (2)
Moderate	1	16	19.9 ± 2.9	10 M, 6 F	56.3 ± 10.8	34.6 ± 7.8	42.3 ± 4.0	4 (1), 6 (2), 2 (3), 4 (4)
	2	18	22.8 ± 4.5	13 M, 5 F	53.6 ± 9.7	31.8 ± 5.7	42.7 ± 3.8	8 (1), 5 (2), 3 (3), 2 (4)
	3	16	21.6 ± 4.7	13 M, 3 F	54.5 ± 13.9	35.2 ± 5.2	43.6 ± 2.8	5 (1), 6 (2), 3 (3), 2 (4)
Severe	1	14	72.9 ± 35.0	11 M, 3 F	45.5 ± 10.5	39.1 ± 10.1	44.3 ± 4.2	2 (1), 3 (2), 5 (3), 4 (4)
	2	16	66.6 ± 29.6	13 M, 3 F	50.2 ± 11.0	40.1 ± 8.5	46.6 ± 3.2	1 (1), 6 (2), 5 (3), 4 (4)
	3	10	69.6 ± 39.1	6 M, 4 F	51.9 ± 12.2	40.2 ± 7.5	43.8 ± 3.5	2 (1), 4 (2), 4 (3)

**Table 3:** Structure–Function–Symptom Interpretation of Dominant Acoustic Features Across OSA Severity Comparisons.

Severity Model	Dominant Feature Types	Structure–Function–Symptom Interpretation
Non-OSA vs. Mild-OSA	Spectral skewness of the power spectrum and texture uniformity of the bispectrum	Mild structural airway compliance and early narrowing (Structure) introduce intermittent airflow instability (Function), producing subtle turbulence and disrupted nonlinear coupling that manifest clinically (Symptom) as early increases in AHI.
Non-OSA vs. Moderate-OSA	Spectral power from the power spectrum and fragmentation of bispectral patterns	Progressive anatomical narrowing and reduced airway stiffness (Structure) generate sustained turbulent airflow and fragmented nonlinear interactions (Function), corresponding clinically (Symptom) to increased AHI and frequent obstructive events.
Non-OSA vs. Severe-OSA	Energy, complexity, and impulsiveness derived from the power spectrum and bispectrum	Severe upper-airway collapsibility and loss of neuromuscular control (Structure) result in chaotic, high-energy airflow and impulsive breathing sounds (Function), which clinically manifest (Symptom) as severe OSA with high AHI.
Mild-OSA vs. Moderate-OSA	Bandwidth expansion in the power spectrum and texture irregularity in the bispectrum	Worsening airway compromise (Structure) shifts airflow from intermittent to persistent instability (Function), reflected clinically (Symptom) by escalating AHI and sustained breathing disruption.
Mild-OSA vs. Severe-OSA	Entropy, Root Mean Square (RMS), and high-frequency variability of power spectral and bispectral representations	Dominant structural airway vulnerability (Structure) overwhelms compensatory mechanisms, producing highly irregular and energetic airflow (Function) that manifests clinically (Symptom) as severe OSA.
Moderate-OSA vs. Severe-OSA	Variability and topological complexity of power spectral and bispectral structures	Further loss of airway resilience and increased collapsibility (Structure) lead to chaotic airflow dynamics and extreme variability (Function), clinically reflected (Symptom) by markedly elevated AHI.

**Table 4:** Top 10 Ranked Tracheal Breathing Sound Features for OSA Severity Classification (Lower is better).

Feature Name	Average Rank by Corr	Average Rank by SHAP	Overall Average Rank
Average_BBox_Spectral	1	1	1
MouthInspiration_ConnectedComponents	1	2	1.5
MouthExpiration_FreqCentroid	2	1	1.5
Average_BBox_SpectralFlux	2	1	1.5
MouthExpiration_Range_SCBW_Bandwidth	1	2	1.5
Average_BBox_CentroidY	3	1	2
MouthInspiration_Range_MeanPower	3	1	2
Average_BBox_Entropy	3	2	2.5
Average_BBox_TextureEnergy	3	3	3
NoseExpiration_Range_FreqSkewness	3	3	3

**Table 5:** Top 10 Most Stable Tracheal Breathing Sound Features. Lower Absolute Delta AUC indicates higher feature stability across cross-validation folds, while Absolute Delta Correlation reflects the consistency of feature correlation. Features are considered stable/significant if Abs Delta AUC ≤ 0.01 and Abs Delta Correlation ≤ 0.1, reflecting minimal variation and robust performance across folds. Features with Abs Delta Correlation between 0.1 and 0.15 are considered slightly unstable but still statistically acceptable, as this slight variation does not substantially affect their overall predictive reliability.

Feature Name	Abs Delta AUC	Abs Delta Correlation
Average_BBox_Skewness	0.001231	0.005491
MouthExpiration_CoefVariation	0.001806	0.137229
Average_BBox_FrequencyBandwidth	0.002441	0.09704
Average_BBox_FrequencyBandwidth	0.002511	0.070444
Average_BBox_TextureEnergy	0.002917	0.076107
MouthExpiration_Perimeter	0.002917	0.0041
MouthExpiration_FrequencyBandwidth	0.002917	0.016699
Average_BBox_Median	0.003554	0.113319
Average_BBox_FrequencyBandwidth	0.003667	0.152313
MouthInspiration_Range_FreqSkewness	0.003667	0.038156

**Table 6:** Top 10 Pearson correlations between breathing sound features and anthropometric measures (NC, BMI, Sex, MPS). Each row indicates which feature is correlated with which anthropometric variable, along with the fold and severity comparison.

Feature Name	Anthropometric Feature	Pearson Correlation	Fold	Comparison
MouthExpiration_Peak	NC	1	2	Mild-OSA vs. Severe-OSA
Average_BBox_TextureHomogeneity	Sex	0.9998	1	Non-OSA vs. Moderate-OSA
MouthExpiration_Entropy	BMI	0.999	2	Mild-OSA vs. Severe-OSA
Average_BBox_Mean	Sex	0.9987	1	Non-OSA vs. Mild-OSA
Average_BBox_IQR	Sex	0.9985	3	Mild-OSA vs. Moderate-OSA
MouthInspiration_FreqCentroid	BMI	0.9985	3	Mild-OSA vs. Severe-OSA
Average_BBox_Energy	NC	0.9985	2	Non-OSA vs. Severe-OSA
Average_BBox_AspectRatio	MPS	0.9983	1	Non-OSA vs. Severe-OSA
MouthExpiration_FreqCentroid	MPS	0.9982	2	Mild-OSA vs. Moderate-OSA
Average_BBox_CentroidY	MPS	0.9982	3	Mild-OSA vs. Moderate-OSA

**Table 7:** Comparison of the proposed framework with representative wakefulness-based OSA screening modalities. Acc: accuracy, Sens: sensitivity, Spec: specificity.

Modality	Reference	Dataset Size	Task	Performance Metrics	Uncertainty Modeling
Questionnaires	[60]	9206 (Meta-analysis)	Screening	Sens: ~90–96%	No
				Spec: ~25–34%	
	[65]	234 (Clinical)	Screening	Sens: ~95%	No
				Spec: ~5%	
Facial Analysis	[61]	365	Binary	Acc: 69.8%	No
				Sens: 68.5%	
				Spec: 76.4%	
	[66]	180	Binary	Acc: 79.4%	No
				Sens: 69.7%	
Speech Analysis	[67]	40 OSA/40 Control	Binary	Acc: 81.0%	No
				Sens: 77.5%	
				Spec: 85.0%	
	[68]	190 (AHI < 15) and 208 (AHI > 15)	Binary	Acc: 77.1%	No
				Sens: 75.0%	
				Spec: 79.0%	
Pharyngometry	[63]	46 (AHI < 5) and 254 (AHI > 15)	Binary	Sens: 97.6%	No
				Spec: 100%	
NEP	[62]	24 (AHI < 5) and 24 (AHI > 30)	Binary	Sens: 91.7%	No
				Spec: 95.8%	
Tracheal Sound	[12]	109 (AHI < 15) and 90 (AHI > 15)	Binary	Acc: 81.4%	No
				Sens: 80.9%	
				Spec: 82.1%	
	[16]	17 (AHI < 5) and 35 (AHI > 5)	Binary	Acc: 83.3%	No
				Sens: 85.0%	
				Spec: 81.3%	
Proposed	Propsed	74 Non-OSA, 35 Mild, 50 Moderate, and 40 Severe	Multi-class	AUC Range: 0.86–0.97	Yes (Bootstrap Aggregation & 95% CI)

## Data Availability

The data presented in this study are available on request from the PI of the study (last author) due to ethical and consent-related restrictions, as the datasets contain human audio recordings and can only be shared under controlled access following approval of a signed data-use consent agreement.

## Abbreviations

The following abbreviations are used in this manuscript:

AHI	Apnea–Hypopnea Index
AUC	Area Under the Receiver Operating Characteristic Curve
AbsDeltaAUC	Absolute Change in AUC
BBox	Bounding Box
BMI	Body Mass Index
CV	Cross-Validation
CQT	Constant-Q Transform
FM	Frequency Mean
FM2M	Mean Frequency Ratio
HP	Harmonic–Percussive
IQR	Interquartile Range
k-Fold CV	K-Fold Cross-Validation
MFCC	Mel-Frequency Cepstral Coefficients
ML	Machine Learning
NC	Neck Circumference
NEP	Negative Expiratory Pressure
OSA	Obstructive Sleep Apnea
RFE	Recursive Feature Elimination
ROC	Receiver Operating Characteristic
RQA	Recurrence Quantification Analysis
RMS	Root Mean Square
SD	Standard Deviation
SHAP	Shapley Additive exPlanations
SNR	Signal-to-Noise Ratio
TBS	Tracheal Breathing Sounds
ΔAUC	Change in AUC

## Appendix A. Top Selected Features for Each Model

**Table A1:** Summary of Top Selected Features for Each Model.

Feature Number	Non-OSA vs. Mild-OSA	Non-OSA vs. Moderate-OSA	Non-OSA vs. Severe-OSA	Mild-OSA vs. Moderate-OSA	Mild-OSA vs. Severe-OSA	Moderate-OSA vs. Severe-OSA
1	MouthExpiration_Range_SpectralEntropy	Average_Range_SpectralSkewness	NoseInspiration_Range_SpectralKurtosis	NoseExpiration_Range_MeanPower	MouthExpiration_Range_SCBW_Bandwidth	NoseExpiration_Range_SpectralCrest
2	MouthExpiration_Range_SpectralCrest	NoseInspiration_Range_SpectralSkewness	NoseInspiration_Range_FreqSkewness	MouthExpiration_IQR	Average_Average_BBoxes_EnFBiD	Average_BBox_NumHoles
3	MouthExpiration_Range_SpectralCrest	Average_BBox_Entropy	NoseExpiration_Range_SCBW_Bandwidth	MouthInspiration_MFCCMean	NoseExpiration_BBox_Range	MouthInspiration_WaveletApproxEntropy
4	NoseInspiration_Range_SCBW_Bandwidth	MouthInspiration_Range	Average_BBox_TextureEnergy	NoseExpiration_WaveletApproxSkewness	Average_Range_SpectralCrest	NoseExpiration_PeakCount
5	MouthInspiration_MFCCMean	MouthExpiration_WaveletApproxSpectralBandwidth	Average_BBox_CentroidY	MouthInspiration_MFCCMean	NoseInspiration_BBox_Median	MouthInspiration_MFCCMean
6	MouthInspiration_MFCCMedian	MouthExpiration_WaveletDetailEntropy	NoseInspiration_BBox_AspectRatio	MouthInspiration_MFCCStd	NoseInspiration_BBox_PeakValue	MouthExpiration_Range_SCBW_Bandwidth
7	NoseExpiration_WaveletApproxMean	MouthInspiration_IQR	MouthInspiration_BBox_EulerNumber	MouthInspiration_SpectralCentroid	NoseInspiration_BBox_FreqCentroid	MouthInspiration_Range_RMS
8	NoseExpiration_Range_BandPower	NoseInspiration_CoefVariation	MouthInspiration_MeanValue	MouthInspiration_SpectralBandwidth	NoseInspiration_BBox_PeakValue	NoseInspiration_Range_MeanPower
9	NoseExpiration_Range_SpectralSkewness	NoseExpiration_BBox_Entropy	MouthInspiration_StdValue	MouthInspiration_MFCCKurtosis	NoseInspiration_BBox_FreqCentroid	NoseInspiration_Range_SCBW_Bandwidth
10	MouthInspiration_BBox_NumHoles	Average_DIR_Histogram	MouthExpiration_TotalEnergy	MouthExpiration_WaveletSpectralCentroid	NoseInspiration_BBox_SpectralFlux	NoseInspiration_Range_SpectralEnergy
11	MouthExpiration_BBox_FreqCentroid	Average_AVP_Histogram	MouthExpiration_NormalizedEnergy	MouthExpiration_CQTBandwidthDynamicRange	NoseInspiration_BBox_ConnectedComponents	NoseInspiration_Range_BandPower
12	MouthExpiration_BBox_Kurtosis	MouthInspiration_ZeroCrossing	MouthExpiration_StdAbsBi	MouthExpiration_MFCCStd	NoseInspiration_BBox_EulerNumber	MouthInspiration_BBox_RegionArea
13	MouthExpiration_BBox_Area	MouthInspiration_WaveletDetailSpectralBandwidth	MouthExpiration_SymStd	MouthExpiration_SpectralCentroid	NoseInspiration_BBox_IQR	NoseInspiration_BBox_IQR
14	MouthExpiration_BBox_Diagonal	MouthInspiration_WaveletDetailSpectralBandwidth	MouthExpiration_Perimeter	MouthExpiration_MFCCSkewness	NoseInspiration_BBox_FreqCentroid	NoseInspiration_BBox_TextureHomogeneity
15	MouthExpiration_BBox_NumHoles	MouthInspiration_MFCCMean	MouthExpiration_CentroidY	MouthExpiration_MFCCKurtosis	NoseInspiration_BBox_BBoxArea	NoseInspiration_BBox_NumHoles
16	MouthExpiration_BBox_AspectRatio	MouthInspiration_MFCCKurtosis	MouthExpiration_Perimeter	MouthExpiration_MFCCKurtosis	NoseInspiration_BBox_FreqCentroid	NoseExpiration_BBox_TextureContrast
17	MouthExpiration_BBox_TextureContrast	MouthInspiration_PBP_Skewness	MouthExpiration_CentroidX	NoseInspiration_ZeroCrossing	NoseInspiration_BBox_TextureEnergy	NoseExpiration_BBox_AspectRatio
18	NoseInspiration_BBox_Diagonal	MouthInspiration_TP_Histogram	NoseInspiration_Average_BBox_EnFBiD	NoseInspiration_WaveletApproxSkewness	NoseInspiration_BBox_EulerNumber	NoseExpiration_BBox_Perimeter
19	NoseInspiration_BBox_TextureEnergy	MouthInspiration_TP_MaxProb	NoseInspiration_Average_BBox_MeanBiDF	NoseInspiration_WaveletApproxSpectralCentroid	NoseInspiration_BBox_StdValue	Average_WaveletDetail_MaxToMinRatio
20	Average_WaveletApproxMaxToMinRatio	MouthInspiration_EP_MaxEnergy	NoseInspiration_Average_BBox_WCOBDFx	NoseInspiration_WaveletDetailKurtosis	NoseInspiration_BBox_Entropy	Average_WaveletDetail_Kurtosis
21	NoseInspiration_HurstExponent	MouthExpiration_WaveletApproxKurtosis	NoseInspiration_Average_BBox_WCOBDFy	NoseInspiration_CQTStdPower	NoseInspiration_BBox_Entropy	MouthInspiration_LyapunovExponentMean
22	NoseExpiration_KatzFD	MouthExpiration_WaveletApproxSkewness	NoseInspiration_Average_BBox_Hf1	NoseInspiration_CQTSkewnessPower	NoseInspiration_BBox_TextureContrast	MouthInspiration_BandPowerHigh
23	MouthInspiration_Range_PeakFrequency	MouthExpiration_PBP_Kurtosis	NoseInspiration_Average_BBox_Hf2	NoseInspiration_CQTTemporalCentroid	NoseInspiration_BBox_TextureEnergy	NoseInspiration_WaveletApproxEntropy
24	MouthExpiration_BBox_CentroidY	MouthExpiration_PBP_Entropy	NoseInspiration_Average_BBox_Reserved	NoseInspiration_CQTSpectralCentroid	NoseInspiration_BBox_StdValue	NoseInspiration_WaveletApproxEntropy
25	MouthExpiration_BBox_PeakValue	MouthInspiration_Range_FM2MFreq	NoseInspiration_Average_BBox_EnFBiD	NoseInspiration_CQTBandwidthDynamicRange	NoseExpiration_BBox_ConnectedComponents	NoseInspiration_WaveletDetailSpectralCentroid
26	MouthExpiration_BBox_TextureCorrelation	MouthInspiration_Range_MeanPower	NoseInspiration_Average_BBox_MeanBiDF	NoseInspiration_MFCCSkewness	NoseExpiration_BBox_Median	NoseExpiration_WaveletApproxSkewness
27	NoseInspiration_BBox_Range	MouthInspiration_Range_RMS	NoseInspiration_Average_BBox_WCOBDFx	NoseInspiration_MFCCKurtosis	NoseExpiration_BBox_IQR	Average_AVP_Mean
28	NoseInspiration_BBox_Std	MouthInspiration_Range_FM2MFreq	NoseInspiration_Average_BBox_WCOBDFy	NoseInspiration_SpectralBandwidth	NoseExpiration_BBox_TextureCorrelation	MouthInspiration_KatzFD
29	NoseExpiration_BBox_FractalDimension	MouthInspiration_Range_BandPower	NoseInspiration_Average_BBox_Hf1	NoseInspiration_MFCCKurtosis	NoseExpiration_BBox_TextureEnergy	MouthInspiration_LyapunovExponentMax
30	NoseExpiration_BBox_Std	MouthInspiration_Range_Std	NoseInspiration_Average_BBox_Hf2	NoseInspiration_TP_Skewness	NoseExpiration_BBox_FractalDimension	MouthExpiration_MFCCMedian
31	MouthExpiration_MFCCMedian	MouthInspiration_Range_FM2MFreq	NoseInspiration_Average_BBox_Reserved	NoseExpiration_WaveletDetailSkewness	NoseExpiration_BBox_ConnectedComponents	NoseInspiration_WaveletDetailEntropy
32	MouthExpiration_PBP_Kurtosis	MouthInspiration_Range_FM2MFreq	NoseInspiration_Average_BBox_BisEntropy	NoseExpiration_CQTMeanPower	NoseExpiration_BBox_CentroidY	NoseInspiration_CQTGaborEnergyMean
33	MouthExpiration_TP_Histogram	MouthInspiration_Range_FM2MFreq	NoseInspiration_BBox_SpectralFlux	NoseExpiration_CQTSkewnessPower	NoseExpiration_BBox_Compactness	NoseInspiration_MFCCMedian
34	MouthExpiration_TP_MaxProb	MouthExpiration_Range_Std	NoseInspiration_BBox_ConnectedComponents	NoseExpiration_CQTSpectralDynamicsStd	NoseExpiration_BBox_Energy	Average_Range_Maximum
35	MouthExpiration_TP_Ratio	MouthExpiration_Range_Std	NoseInspiration_BBox_Skewness	MouthInspiration_ZeroCrossing	NoseExpiration_BBox_FreqCentroid	Average_Range_SCBW_Bandwidth

## Appendix B. Analysis of Clinical Interpretation of Top Features for All Models

### Appendix B.1. Non-OSA vs. Mild-OSA

- **MouthInspiration_Range_FreqSkewness:** Measures asymmetry of the power spectrum during mouth inspiration within the relevant frequency range. Positive values indicate dominance of lower frequencies, negative values indicate dominance of higher frequencies. Reflects shifts in frequency energy distribution due to airflow changes, turbulence, or airway geometry.
- **Average_BBox_TextureEnergy:** Indicates uniformity of bispectral texture. Higher energy suggests consistent patterns, while lower values indicate heterogeneous local coupling. In OSA, irregular airflow can produce less uniform bispectral coupling patterns, consistent with more complex or intermittent nonlinear flow-tissue interactions.
- **Average_BBox_FrequencyCentroidX:** Centroid of bispectral energy along the f1-axis. Shifts in this centroid indicate changes in dominant coupling frequency, reflecting airflow-tissue interactions or turbulence associated with airway narrowing.
- **Average_BBox_TextureCorrelation:** Measures gray-level co-occurrence texture correlation. Higher values indicate structured bispectral patterns, suggesting stable nonlinear coupling. Reduced correlation reflects heterogeneous coupling, consistent with variable airflow instability.
- **Average_BBox_Perimeter:** Perimeter of high-intensity regions within the bispectral bounding box. Larger perimeter suggests fragmented coupling, possibly indicating variable airflow instability.
- **Average_BBox_TextureHomogeneity:** Measures uniformity of gray-level values. Higher values indicate smoother patterns; lower values indicate irregular textures, reflecting turbulent airflow.
- **Average_BBox_IQRValue:** Range between the first and third quartiles of bispectral intensity. Larger IQR indicates greater variability in the central portion of the sound signal, potentially associated with airflow turbulence.
- **Average_BBox_Compactness:** How compact high-intensity regions are. Less compact patterns suggest more diffuse sound energy, consistent with irregular airflow.
- **MouthInspiration_Range_SpectralSkewness:** Asymmetry of the power spectrum during mouth inspiration within a relevant frequency range. Changes may indicate airflow limitation or obstruction.

### Appendix B.2. Non-OSA vs. Moderate-OSA

- **Average_Range_Maximum:** Highest amplitude within the relevant frequency range, representing peak breathing sound energy. Higher values may indicate more turbulent airflow due to airway narrowing.
- **Average_BBox_BoundingBoxDiagonal:** Length of the bounding box diagonal for a bispectral patch. Larger diagonal implies broader distribution of nonlinear interactions, consistent with more complex flow disturbances in moderate OSA.
- **Average_Range_MeanPower:** Average spectral energy across the relevant frequency range. Reflects overall intensity of breathing sounds in that band.
- **Average_BBox_ConnectedComponents:** Counts distinct high-intensity regions in the bispectral patch. More regions suggest fragmented coupling, potentially due to multiple flow structures or vibration sites.
- **Average_BBox_TextureContrast:** Local contrast differences in bispectral patches. Higher contrast indicates stronger differences between regions of coupling, compatible with intermittent obstructions.
- **Average_BBox_FrequencyCentroidY:** Centroid of bispectral energy along the f2-axis. Shifts indicate changes in dominant coupling frequencies, reflecting altered airflow-tissue interactions.
- **Average_Range_BandPower:** Total spectral power within a relevant mid-frequency range. Higher power may indicate stronger turbulent airflow.
- **Average_Range_MeanPower:** Average spectral energy, indicative of airflow consistency.
- **Average_Range_RMS:** Standard amplitude measure within the frequency range. Higher RMS indicates more variable or intense breathing sounds, associated with stronger flow disturbances.

### Appendix B.3. Non-OSA vs. Severe-OSA

- **Average_BBox_MeanValue:** Average amplitude or intensity of the sound signal. In OSA, increased respiratory effort produces louder, higher-amplitude sounds.
- **Average_BBox_TextureEnergy:** Uniformity of bispectral texture. Lower energy indicates more heterogeneous local coupling, reflecting complex airflow-tissue interactions.
- **MouthInspiration_Range_FreqSkewness:** Asymmetry of the power spectrum during mouth inspiration within the relevant frequency range. Positive values indicate low-frequency dominance, negative values high-frequency dominance.
- **Average_BBox_FractalDimension:** Complexity or irregularity of local patterns. Higher values indicate more intricate structures, compatible with chaotic airflow in severe OSA.
- **Average_BBox_MedianValue**: Median local intensity; less sensitive to outliers than the mean. Changes reflect shifts in breathing sound intensity.
- **Average_BBox_EnergyValue:** Total energy within the bounding box. Higher energy indicates stronger, more intense breathing sounds, often associated with airway narrowing.
- **Average_BBox_EulerNumber:** Topological feature reflecting the number of connected components minus holes. Indicates complexity of sound events, altered by intermittent airflow or airway vibrations.
- **Average_BBox_AspectRatio:** Width-to-height ratio of detected sound events; changes may reflect altered spectral characteristics due to airway dynamics.
- **Average_BBox_ConnectedComponents:** Counts distinct high-intensity regions; more regions suggest fragmented coupling due to multiple airflow structures.
- **Average_BBox_KurtosisValue:** Measures tailedness of the sound signal distribution. Higher kurtosis indicates sudden, sharp sound events associated with airway collapse or reopening.

### Appendix B.4. Mild-OSA vs. Moderate-OSA

- **MouthExpiration_BBox_AspectRatio:** Width-to-height ratio during mouth expiration; indicates alterations in spectral characteristics due to airway dynamics.
- **Average_BBox_MedianValue:** Median local intensity. Changes reflect general shifts in breathing sound intensity.
- **Average_BBox_TextureEnergy:** Uniformity of sound texture; lower values suggest heterogeneous patterns due to turbulent airflow.
- **MouthExpiration_Range_FM2MFreq:** Ratio of frequency-modulated to mean spectral energy in a relevant range; reflects redistribution of low-frequency energy, indicating airflow variations.
- **Average_BBox_FrequencyBandwidthY:** Effective bandwidth along the frequency axis; broader bandwidth suggests wider participation of frequencies, consistent with turbulent airflow.
- **Average_BBox_Perimeter:** Perimeter of high-intensity regions; larger perimeter indicates more fragmented structure, consistent with variable airflow instability.
- **Average_BBox_FrequencyCentroidY:** Centroid of bispectral energy along f2-axis; shifts indicate changes in dominant frequency regions.
- **MouthExpiration_BBox_TextureEnergy:** Uniformity of sound pattern; lower values indicate less consistent sound patterns, reflecting turbulence or intermittent obstruction.

### Appendix B.5. Mild-OSA vs. Severe-OSA

- **MouthInspiration_BBox_FrequencyCentroidX:** Centroid along f1-axis; shifts indicate changes in dominant frequency region, reflecting airflow dynamics.
- **Average_Average_BBoxes_Entropy:** Overall entropy across bounding boxes; higher values indicate irregular airflow or turbulent sounds in severe OSA.
- **Average_Range_RMS:** Standard amplitude within the relevant frequency range; higher RMS indicates variable or stronger breathing sound intensity.
- **Average_BBox_EnergyValue:** Total energy in bounding box; higher energy reflects increased respiratory effort or airway narrowing.
- **MouthInspiration_Range_StandardDeviation:** Variability of sound amplitude; higher SD indicates greater fluctuation in airflow or turbulence.
- **Average_Range_FM2MFreq:** Low-frequency energy ratio; reflects subtle airflow variations or airway patency changes.
- **MouthInspiration_BBox_TextureContrast:** Local contrast; higher values indicate strong differences between adjacent sound regions.
- **MouthExpiration_BBox_FrequencyBandwidthY:** Bandwidth along frequency axis; broader bandwidth indicates more turbulent airflow.
- **Average_Range_StandardDeviation:** Variability of amplitude; higher SD reflects irregular airflow.
- **MouthExpiration_Range_SpectralEnergy:** Total spectral energy within relevant range; higher energy corresponds to stronger, more turbulent airflow.

### Appendix B.6. Moderate-OSA vs. Severe-OSA

- **Average_BBox_MedianValue:** Median intensity; indicates overall breathing sound level.
- **Average_BBox_IQRValue:** Range between first and third quartiles; larger IQR suggests greater variability, linked to turbulence.
- **Average_BBox_EnergyValue:** Total energy; higher values reflect stronger, more intense breathing sounds due to airflow restriction.
- **Average_BBox_RangeValue:** Difference between maximum and minimum values; larger range indicates pronounced variations in sound intensity.
- **Average_BBox_KurtosisValue:** Measures tailedness; higher kurtosis indicates sudden peaks in sound, linked to airway collapse or reopening.
- **Average_BBox_Compactness:** How compact high-intensity regions are; less compact indicates more diffuse sound energy due to turbulent airflow.
- **Average_BBox_StdValue:** Dispersion of sound values; higher SD indicates greater variability, reflecting irregular airflow.
- **Average_BBox_IQRValue:** Central variability of sound; larger values indicate more turbulence.
- **Average_BBox_EulerNumber:** Complexity of sound events; reflects structure or continuity of airflow events.
- **Average_BBox_EntropyValue:** Randomness or unpredictability of sound; higher entropy indicates more chaotic or turbulent airflow.

## Appendix C. Analysis for Non-OSA vs. Mild-OSA

**Table A2:** Top 10 ranked features for Non-OSA vs. Mild-OSA classification based on AUC Test and correlation performance.

Feature Name	AUC Test	Corr Test
MouthInspiration_Range_FreqSkewness	0.787037	0.498762
Average_BBox_TextureEnergy	0.666667	0.301309
Average_BBox_FrequencyCentroidX	0.657407	0.145422
Average_BBox_TextureCorrelation	0.657407	0.145286
Average_BBox_Perimeter	0.657407	0.0772519
Average_BBox_TextureEnergy	0.644444	0.0914112
Average_BBox_TextureHomogeneity	0.638889	0.0837961
Average_BBox_IQRValue	0.62963	0.211449
Average_BBox_Compactness	0.62963	0.211449
MouthInspiration_Range_SpectralSkewness	0.62963	0.237871

**Table A3:** Top 10 most stable features based on absolute change in AUC (AbsDeltaAUC) for Non-OSA vs. Mild-OSA classification.

Feature Name	Abs Delta AUC
MouthInspiration_Range_SpectralEntropy	0.0102916
Average_BBox_TextureEnergy	0.0241135
Average_BBox_FrequencyCentroidY	0.0441738
Average_BBox_SpectralFlux	0.0498553
Average_BBox_EntropyValue	0.0613248
Average_BBox_BoundingBoxDiagonal	0.0613248
Average_BBox_Compactness	0.0627066
Average_BBox_SpectralFlux	0.0728632
Average_BBox_FrequencyCentroidX	0.0728632
Average_BBox_FractalDimension	0.0860684

**Table A4:** Top 10 correlations between selected features and anthropometric characteristics in Non-OSA vs. Mild-OSA participants.

Feature Name	Anthropometric Feature	Pearson Correlation
Average_BBox_MeanValue	Sex	0.998692
Average_BBox_NumHoles	MPS	0.997553
Average_BBox_FrequencyCentroidX	MPS	0.997288
Average_BBox_StdValue	Sex	0.997255
Average_BBox_CoefVariation	Sex	0.997255
Average_BBox_Perimeter	Sex	0.99691
Average_BBox_MeanValue	MPS	0.994605
Average_BBox_Compactness	NC	0.994605
Average_BBox_TextureContrast	NC	0.993768
Average_BBox_NumHoles	NC	0.993507

**Table A5:** Top 10 features showing the highest Pearson correlations with AHI in Non-OSA vs. Mild-OSA comparison.

Feature Name	Pearson Correlation
Average_BBox_MeanValue	0.994605
Average_BBox_Compactness	0.994605
Average_BBox_AspectRatio	0.984433
Average_BBox_Compactness	0.984433
Average_BBox_MedianValue	0.978554
Average_BBox_FrequencyCentroidY	0.973494
Average_BBox_FrequencyBandwidthY	0.969688
MouthExpiration_Range_SCBW_Bandwidth	0.969226
Average_BBox_TextureContrast	0.960109
Average_BBox_TextureEnergy	0.951338

## Appendix D. Analysis for Non-OSA vs. Moderate-OSA

**Table A6:** Top 10 ranked features for Non-OSA vs. Moderate-OSA classification based on AUC Test and correlation performance.

Feature Name	AUC Test	Corr Test
MouthInspiration_Range_FreqSkewness	0.787037	0.498762
Average_Range_Maximum	0.849794	0.458931
Average_BBox_BoundingBoxDiagonal	0.81893	0.537497
Average_Range_MeanPower	0.816872	0.4697
Average_BBox_ConnectedComponents	0.792181	0.400149
Average_BBox_TextureContrast	0.788066	0.421496
Average_BBox_BoundingBoxDiagonal	0.781893	0.479976
Average_BBox_FrequencyCentroidY	0.76749	0.336108
Average_Range_BandPower	0.738683	0.437269
MouthInspiration_Range_FreqSkewness	0.738683	0.436397

**Table A7:** Top 10 most stable features based on absolute change in AUC (AbsDeltaAUC) for Non-OSA vs. Moderate-OSA classification.

Feature Name	AbsDeltaAUC
Average_BBox_FrequencyBandwidthX	0.00244068
Average_BBox_RangeValue	0.00810185
Average_BBox_AspectRatio	0.015558
Average_BBox_EnergyValue	0.0266176
Average_BBox_MeanValue	0.0277022
Average_BBox_EulerNumber	0.0319368
Average_BBox_KurtosisValue	0.0366769
Average_BBox_RangeValue	0.0470624
Average_BBox_BoundingBoxArea	0.0470624
Average_BBox_FrequencyBandwidthX	0.047544

**Table A8:** Top 10 correlations between selected features and anthropometric characteristics in Non-OSA vs. Moderate-OSA participants.

Feature Name	Anthropometric Feature	Pearson Correlation
Average_BBox_TextureHomogeneity	Sex	0.999783
Average_BBox_RangeValue	BMI	0.997506
NoseExpiration_Range_MeanPower	Smoke History	0.996784
Average_BBox_FrequencyCentroidY	NC	0.995961
Average_BBox_BoundingBoxDiagonal	Smoke History	0.990975
Average_BBox_TextureHomogeneity	MPS	0.989529
Average_BBox_IQRValue	NC	0.987245
Average_BBox_TextureHomogeneity	Age	0.982519
Average_BBox_MeanValue	Sex	0.982193
Average_BBox_ConnectedComponents	Smoke History	0.980487

**Table A9:** Top 10 features showing the highest Pearson correlations with AHI in Non-OSA vs. Moderate-OSA comparison.

Feature Name	Pearson Correlation
Average_BBox_RangeValue	0.962649
Average_BBox_TextureCorrelation	0.96016
Average_BBox_Perimeter	0.930377
Average_BBox_MeanValue	0.917263
Average_BBox_TextureEnergy	0.911276
Average_BBox_FrequencyCentroidX	0.89864
Average_BBox_SkewnessValue	0.882517
Average_BBox_IQRValue	0.877315
Average_BBox_StdValue	0.844572
Average_BBox_FractalDimension	0.735205

## Appendix E. Analysis for Non-OSA vs. Severe-OSA

**Table A10:** Top 10 ranked features for Non-OSA vs. Severe-OSA classification based on AUC Test and correlation performance.

Feature Name	AUC Test	Corr Test
Average_BBox_MeanValue	0.860248	0.505319
Average_BBox_TextureEnergy	0.775463	0.444318
MouthInspiration_Range_FreqSkewness	0.775	0.413793
Average_BBox_FractalDimension	0.73913	0.429386
Average_BBox_MedianValue	0.733333	0.242264
Average_BBox_EnergyValue	0.729167	0.216856
Average_BBox_EulerNumber	0.716667	0.246949
Average_BBox_AspectRatio	0.708333	0.200236
Average_BBox_ConnectedComponents	0.708333	0.215239
Average_BBox_KurtosisValue	0.708333	0.267374

**Table A11:** Top 10 most stable features based on absolute change in AUC (AbsDeltaAUC) for Non-OSA vs. Severe-OSA classification.

Feature Name	AbsDeltaAUC
Average_BBox_SkewnessValue	0.00123128
Average_BBox_FrequencyBandwidthX	0.0025107
Average_BBox_FrequencyBandwidthX	0.00366667
MouthInspiration_Range_FreqSkewness	0.00366667
Average_BBox_Compactness	0.00418763
Average_BBox_FrequencyBandwidthX	0.004408
Average_BBox_KurtosisValue	0.00454831
Average_BBox_SkewnessValue	0.00578492
Average_BBox_EulerNumber	0.00608255
Average_BBox_ConnectedComponents	0.00933082

**Table A12:** Top 10 correlations between selected features and anthropometric characteristics in Non-OSA vs. Severe-OSA participants.

Feature Name	Anthropometric Feature	Pearson Correlation
Average_BBox_EnergyValue	NC	0.998475
Average_BBox_AspectRatio	MPS	0.99834
Average_BBox_StdValue	Age	0.995421
Average_BBox_EulerNumber	Sex	0.995285
Average_BBox_Perimeter	MPS	0.99373
Average_BBox_IQRValue	Sex	0.991446
Average_BBox_NumHoles	Smoke History	0.989047
Average_BBox_ConnectedComponents	Age	0.988745
Average_BBox_EnergyValue	Age	0.988332
Average_BBox_MeanValue	Age	0.987492

**Table A13:** Top 10 features showing the highest Pearson correlations with AHI in Non-OSA vs. Severe-OSA comparison.

Feature Name	Pearson Correlation
Average_BBox_PeakValue	0.895716
Average_BBox_BoundingBoxDiagonal	0.881183
Average_BBox_BoundingBoxDiagonal	0.85574
Average_BBox_Perimeter	0.846486
Average_BBox_KurtosisValue	0.835585
Average_BBox_Perimeter	0.828251
Average_BBox_MeanValue	0.769224
Average_BBox_FrequencyCentroidX	0.759227
Average_BBox_EnergyValue	0.752154
Average_BBox_SkewnessValue	0.734339

## Appendix F. Analysis for Mild-OSA vs. Moderate-OSA

**Table A14:** Top 10 ranked features for Mild-OSA vs. Moderate-OSA classification based on AUC Test and correlation performance.

Feature Name	AUC Test	Corr Test
MouthExpiration_BBox_AspectRatio	0.883333	0.496958
Average_BBox_MedianValue	0.788889	0.451368
Average_BBox_TextureEnergy	0.783333	0.258151
MouthExpiration_Range_FM2MFreq	0.6875	0.187878
Average_BBox_FrequencyBandwidthY	0.671875	0.251735
Average_BBox_FrequencyBandwidthY	0.655556	0.0760239
Average_BBox_Perimeter	0.655556	0.0760239
Average_BBox_FrequencyCentroidY	0.644531	0.221861
MouthExpiration_BBox_TextureEnergy	0.644444	0.220908
Average_BBox_TextureEnergy	0.641667	0.294824

**Table A15:** Top 10 most stable features based on absolute change in AUC (AbsDeltaAUC) for Mild-OSA vs. Moderate-OSA classification.

Feature Name	AbsDeltaAUC
MouthExpiration_BBox_CoefVariation	0.00180556
Average_BBox_TextureEnergy	0.00291667
Average_BBox_FrequencyCentroidY	0.00443837
MouthExpiration_Range_FM2MFreq	0.00593891
Average_BBox_FrequencyBandwidthX	0.00599845
Average_BBox_CentroidY	0.00813298
Average_BBox_FrequencyBandwidthY	0.0124323
Average_BBox_CentroidY	0.0142974
Average_BBox_SkewnessValue	0.0192591
Average_BBox_SkewnessValue	0.0218056

**Table A16:** Top 10 correlations between selected features and anthropometric characteristics in Mild-OSA vs. Moderate-OSA participants.

Feature Name	Anthropometric Feature	Pearson Correlation
Average_BBox_IQRValue	Sex	0.998547
MouthExpiration_FrequencyCentroidX	MPS	0.998212
Average_BBox_CentroidY	MPS	0.99816
MouthExpiration_StdValue	NC	0.997504
Average_BBox_MeanValue	MPS	0.997397
MouthExpiration_FrequencyCentroidX	NC	0.996663
Average_BBox_FrequencyCentroidY	Sex	0.996409
Average_BBox_Perimeter	Age	0.996071
Average_BBox_FrequencyCentroidX	Sex	0.995729
Average_BBox_Perimeter	MPS	0.994929

**Table A17:** Top 10 features showing the highest Pearson correlations with AHI in Mild-OSA vs. Moderate-OSA comparison.

Feature Name	Pearson Correlation
Average_BBox_TextureHomogeneity	0.990363
Average_BBox_BoundingBoxDiagonal	0.984096
Average_BBox_EntropyValue	0.9703
Average_BBox_EntropyValue	0.964663
Average_BBox_FrequencyBandwidthX	0.962357
Average_BBox_CentroidY	0.962357
Average_BBox_FrequencyCentroidX	0.956978
Average_BBox_MedianValue	0.953038
Average_BBox_NumHoles	0.952526
Average_BBox_TextureEnergy	0.951101

## Appendix G. Analysis for Mild-OSA vs. Severe-OSA

**Table A18:** Top 10 ranked features for Mild-OSA vs. Severe-OSA classification based on AUC Test and correlation performance.

Feature Name	AUC Test	Corr Test
MouthInspiration_FrequencyCentroidX	0.844444	0.157994
Average_BBox_EnTBiD	0.777778	0.483544
Average_Range_RMS	0.744444	0.67878
Average_BBox_EnergyValue	0.733333	0.513402
MouthInspiration_Range_StdDev	0.722222	0.776042
Average_Range_FM2MFreq	0.714286	0.572164
MouthInspiration_TextureContrast	0.711111	0.380311
MouthExpiration_FrequencyBandwidthY	0.69375	0.214055
Average_Range_StdDev	0.688889	0.621574
MouthExpiration_Range_SpectralEnergy	0.688889	0.516899

**Table A19:** Top 10 most stable features based on absolute change in AUC (AbsDeltaAUC) for Mild-OSA vs. Severe-OSA classification.

Feature Name	AbsDeltaAUC
MouthExpiration_Perimeter	0.00291667
MouthExpiration_FrequencyBandwidthX	0.00291667
MouthInspiration_PeakValue	0.0041751
Average_BBox_NumHoles	0.0042735
MouthExpiration_Perimeter	0.00583333
MouthInspiration_ConnectedComponents	0.00600058
MouthExpiration_CentroidX	0.00666667
MouthExpiration_NumHoles	0.0075
MouthExpiration_PeakValue	0.0075
MouthInspiration_StdValue	0.00816946

**Table A20:** Top 10 correlations between selected features and anthropometric characteristics in Mild-OSA vs. Severe-OSA participants.

Feature Name	Anthropometric Feature	Pearson Correlation
MouthExpiration_PeakValue	NC	0.99998
MouthExpiration_EntropyValue	BMI	0.99901
MouthInspiration_FrequencyCentroidX	BMI	0.998535
MouthInspiration_SpectralFlux	BMI	0.997205
MouthExpiration_PeakValue	NC	0.996188
MouthInspiration_CentroidY	MPS	0.996019
MouthExpiration_Range_RMS	NC	0.99593
MouthExpiration_Range_MeanPower	Sex	0.994836
MouthInspiration_EulerNumber	Sex	0.99472
MouthExpiration_Range_BandPower	Sex	0.994715

**Table A21:** Top 10 features showing the highest Pearson correlations with AHI in Mild-OSA vs. Severe-OSA comparison.

Feature Name	Pearson Correlation
MouthInspiration_IQRValue	0.988287
MouthInspiration_EulerNumber	0.986967
MouthInspiration_AspectRatio	0.980666
MouthInspiration_CentroidX	0.965072
MouthInspiration_FrequencyBandwidthX	0.958011
MouthInspiration_RegionArea	0.942624
MouthExpiration_StdValue	0.902562
MouthInspiration_KurtosisValue	0.879297
MouthInspiration_IQRValue	0.875097
MouthInspiration_MedianValue	0.872257

## Appendix H. Analysis for Moderate-OSA vs. Severe-OSA

**Table A22:** Top 10 ranked features for Moderate-OSA vs. Severe-OSA classification based on AUC Test and correlation performance.

Feature Name	AUC Test	Corr Test
Average_BBox_MedianValue	0.71875	0.239624
Average_BBox_IQRValue	0.690972	0.354004
Average_BBox_EnergyValue	0.680556	0.361267
Average_BBox_RangeValue	0.642361	0.211027
Average_BBox_KurtosisValue	0.637153	0.397161
Average_BBox_Compactness	0.615625	0.423041
Average_BBox_StdValue	0.607143	0.121142
Average_BBox_IQRValue	0.607143	0.121142
Average_BBox_EulerNumber	0.59375	−0.0542083
Average_BBox_EntropyValue	0.59375	−0.0327421

**Table A23:** Top 10 most stable features based on absolute change in AUC (AbsDeltaAUC) for Moderate-OSA vs. Severe-OSA classification.

Feature Name	AbsDeltaAUC
Average_BBox_MedianValue	0.00355392
Average_BBox_RegionArea	0.0053935
Average_BBox_FrequencyCentroidX	0.0101997
Average_BBox_IQRValue	0.0132378
MouthInspiration_Range_SpectralEnergy	0.0278361
Average_BBox_KurtosisValue	0.0325428
Average_BBox_PeakValue	0.0373264
Average_BBox_EnergyValue	0.0503472
Average_BBox_CoefVariation	0.0576721
Average_BBox_SpectralFlux	0.0649846

**Table A24:** Top 10 correlations between selected features and anthropometric characteristics in Moderate-OSA vs. Severe-OSA participants.

Feature Name	Anthropometric Feature	Pearson Correlation
Average_BBox_BoundingBoxArea	NC	0.997757
Average_BBox_RangeValue	Sex	0.997111
Average_BBox_NumHoles	Smoke History	0.996614
Average_BBox_Perimeter	Smoke History	0.996614
Average_BBox_FrequencyBandwidthX	Smoke History	0.995328
Average_BBox_RegionArea	NC	0.992481
Average_BBox_EnergyValue	MPS	0.991225
NoseExpiration_Range_SpectralCrest	Age	0.990493
Average_BBox_PeakValue	Sex	0.988759
Average_BBox_Perimeter	Sex	0.987818

**Table A25:** Top 10 features showing the highest Pearson correlations with AHI in Moderate-OSA vs. Severe-OSA comparison.

Feature Name	Pearson Correlation
Average_BBox_RegionArea	0.977981
Average_BBox_ConnectedComponents	0.966506
Average_BBox_TextureHomogeneity	0.937707
Average_BBox_MeanValue	0.937068
Average_BBox_RegionArea	0.925231
Average_BBox_BoundingBoxArea	0.915942
Average_BBox_EnergyValue	0.909118
Average_BBox_CentroidY	0.906971
Average_BBox_FrequencyCentroidX	0.903769
Average_Range_StdDev	0.890411
